# Supplementary material for: Inter e intra-variability of the best ranked teams: A network analysis in male high-level volleyball
Source: PLoS One. 2023 Feb 2;18(2):e0280365. doi: 10.1371/journal.pone.0280365 (PMC9894390; doi:10.1371/journal.pone.0280365)
Supplement: Attachment — Submitted filename: Response to reviewers.docx [file pone.0280365.s002.docx]

**Response to reviewers**

| General Comments | |
| --- | --- |
| 1. Please ensure that your manuscript meets PLOS ONE's style requirements, including those for file naming | We modified all the text as PLOS ONE’s Style. |
| 2. We suggest you thoroughly copyedit your manuscript for language usage, spelling, and grammar. | We have proofread the entire text. |
| 3. Please ensure that you have specified (1) whether consent was informed and (2) what type you obtained (for instance, written or verbal, and if verbal, how it was documented and witnessed). If your study included minors, state whether you obtained consent from parents or guardians. If the need for consent was waived by the ethics committee, please include this information. | Information on ethics committee approval is available in the methods section. |
| 4. Thank you for stating the following in your Competing Interests section:  "NO authors have competing interests"  Please complete your Competing Interests on the online submission form to state any Competing Interests. If you have no competing interests, please state "The authors have declared that no competing interests exist.", as detailed online in our guide for authors at <http://journals.plos.org/plosone/s/submit->now  This information should be included in your cover letter; we will change the online submission form on your behalf. | No authors have competing interests |
| 5. Please upload a copy of Figure S1-S5, to which you refer in your text on page xx. If the figure is no longer to be included as part of the submission please remove all reference to it within the text.  [Note: HTML markup is below. Please do not edit.] | Done |
| Reviewers' comments: | |
| 3. Have the authors made all data underlying the findings in their manuscript fully available?  The PLOS Data policy requires authors to make all data underlying the findings described in their manuscript fully available without restriction, with rare exception (please refer to the Data Availability Statement in the manuscript PDF file). The data should be provided as part of the manuscript or its supporting information, or deposited to a public repository. For example, in addition to summary statistics, the data points behind means, medians and variance measures should be available. If there are restrictions on publicly sharing data—e.g. participant privacy or use of data from a third party—those must be specified. | We have added all data in the submission. |
| - In the introduction the problem must be presented clearly. Despite being referred to in a general way, I suggest that the problem is presented specifically. | We put the problem more clearly. |
| - Does it matter in the sample whether the observed matches correspond to the same stages of the competition? If not, I think it is important to clarify this situation and consider presenting results according to this contextual variable. The observed actions took into account contextual variables (eg opponent level), a fact that can lead to different presentations from those recorded. | The matches observed were of the same opponent level, this information was added directly to the text:  *“A total of 2,743 offensive actions in Complex I were analyzed, from 22 matches of 2018 Men's Volleyball World Championship between the eight best ranked teams in this edition (Poland, Brazil, United States, Serbia, Italy, Russia, France and Netherlands respectively).”* |
| - The variables presented are part of an observational instrument. Did you go through any validation process? | We utilized variables already validated and used in other studies (as referenced in each description). |
| - It should also be mentioned how the training of the observers was carried out.  - What are the procedures for testing inter- and intra-observer reliability? | We followed the reviewer's recommendations directly in the text:  *“The analysis was performed by three volleyball coaches (more than five years of experience as performance analysts), trained by a high-level coach with more than 10 years of practice and with experience with national and regional teams. For this purpose, 5 games were analyzed together from a competition different from the sample (Final phase of the Men's Brazilian Superliga 20/21), and the final game (composed of 5 sets) was reanalyzed after 1 month to verify the intra and inter-observer reliability, thus resolving any doubts. For the final reliability test, 20% of the actions were reanalyzed, which is above the reference value of 10% [47]. Cohen's Kappa values were between 0.89 and 0.98 with the respective standard errors of 0.07 and 0.01 for the intraobserver analysis, and 1 with the standard error equal to 0 for the interobserver analysis. Such values are above the value recommended by the literature, which is 0.75 [48].”* |
| - Reliability values per variable must be presented. | We added directly to the text.  Reliability values for each variable were: Reception Zone (0,95), Reception Effect (0,89); Attack Tempo (0,95), Attack Type (0,98), Attack Zone (1,00), Play Position (1,00), Block Composition (0,98), Block Touch (0,98), and Attack Effect (0,98) |
| - I suggest the presentation of practical implications and the main conclusions of the study | Done |
| It is not clear how the networks have been constructed and the methodological part regarding the analysis needs to be addressed properly. In particular, the authors need to specify what kind of ties they are considering when constructing the network, highlighting what are the advantages of choosing the eigenvector centrality compared of other centrality indices. | Methodological process was better explained directly in the text.  *“Thus, the eigenvector centrality provides the relevant information about which nodes, or here as game variables used, are more influential in the network, taking into account the connectivity from other nodes that are also more central [49, 50], so, the Eigenvector Centrality depends not only on the number of its adjacent nodes, but also their interaction characteristics [40]. Node size were manipulated to highlight the magnitude of the eigenvector measure using the intrinsic units provided by Gephi Software (Between 300 to 1,500 arbitrary units). Edge corresponds the direct relationship between two nodes defined by number of connections, therefore, thicker edges correspond to a greater number of connections between two nodes [51]. Thus, the node size determines the visual variables contrast according to the eigenvector centrality and the edges thickness, in turn, reveals the weight (given by the number) of the connection directly and indirectly between the nodes [10, 24]. In addition, the Modularity Algorithm was used to detect community structure (8 communities with a cohesion value of 0.871) and the "Fruchterman Reigold" distribution (area 100000) was used organizing the nodes with the highest eigenvector centrality in the center of each subnet [52].”* |
| I suggest the authors to resubmit the manuscript after adding a more consistent methodological part. | Following the recommendations of Reviewer #1, we changed and improved the methodological process of every session directly in the text |
| The Introduction does not have a logical sequence of thoughts and the Discussion can be improved according to the studies already carried out. Only with a major review of these two chapters, one could consider accepting the paper. |  |
